# How Should We Treat Severe Malaria in the Setting of Artemisinin Partial Resistance?

**DOI:** 10.4269/ajtmh.26-0514

**Published:** 2026-09-01

**Authors:** Philip J. Rosenthal, Moses R. Kamya, Ruth Namazzi, Chandy C. John

**Affiliations:** ^1^ Department of Medicine, University of California, San Francisco, California, USA;; ^2^ Infectious Diseases Research Collaboration and Department of Medicine, Makerere University, Kampala, Uganda;; ^3^ Department of Pediatrics and Child Health, Makerere University, Kampala, Uganda;; ^4^ Ryan White Center for Pediatric Infectious Diseases and Global Health, Indiana University School of Medicine, Indianapolis, Indiana, USA

## Abstract

Artemisinin partial resistance (ART-R) threatens the control of falciparum malaria, but its impact on the treatment of severe malaria has been unclear. Two recent studies in Uganda have considered associations between PfK13 mutations linked to ART-R and treatment outcomes in children hospitalized with complicated or severe malaria and treated with intravenous artesunate. Children infected with ART-R parasites experienced delayed parasite clearance compared to those infected with drug-sensitive parasites. However, clinical outcomes, including mortality, length of hospital stay, incidence of subsequent febrile illness, and incidence of readmission did not differ between children infected with ART-R or drug-sensitive parasites. Therefore, at present treatment with intravenous artesunate should remain the standard-of-care to treat severe malaria in sub-Saharan Africa, even in the setting of ART-R, although continued study of treatment efficacy is warranted.

Artemisinins are fast-acting antimalarials that have been the standard of care for the treatment of uncomplicated (as part of artemisinin-based combination therapy, ACT) and severe (as intravenous artesunate) falciparum malaria for over two decades. For severe malaria, the rapid action of artemisinins is likely critical, and two large randomized trials in Asia (mostly adults)^1^ and Africa (children)^2^ showed markedly improved survival after treatment with parenteral artesunate compared to quinine. Intravenous artesunate has since been the standard-of-care to treat severe malaria.^3^

The efficacy of artemisinins to treat severe malaria is now threatened by artemisinin partial resistance (ART-R). ART-R was first identified in Southeast Asia in 2008^4^^,^^5^ and is marked by delayed parasite clearance after treatment of malaria with artemisinin-based therapies.^6^ The impact of ART-R on clinical responses to ACTs for uncomplicated malaria appears to be modest unless resistance to ACT partner drugs is also established; in that case, as seen with dihydroartemisinin-piperaquine in Cambodia, widespread treatment failures can be seen.^7^ Impacts of ART-R may be greater for severe malaria, for which rapid clearance of infecting parasites is vital.

ART-R is associated with any of ∼20 validated PfK13 propeller domain mutations, which were first identified in Southeast Asia.^8^ Some of these variants have since emerged and spread in multiple foci in eastern Africa.^9^ Different ART-R-mediating mutations are seen in different areas of Africa, and key mutations have been associated with delayed parasite clearance after therapy and in laboratory assays for ART-R.^9^

With emergence and spread of ART-R, of particular concern is its impact on the treatment of severe malaria. This concern has been relatively small in southeast Asia, where malaria is uncommon, with few cases of severe malaria. But, the situation is very different in sub-Saharan Africa, where ∼95% of the hundreds of millions of cases and ∼600,000 deaths estimated to be caused by falciparum malaria each year occur.^10^ In rural Africa, children with severe malaria often present late for care, with severe anemia, cerebral malaria, acute kidney injury, and other serious consequences that can rapidly progress to death. There has been concern that clearance of infections with ART-R parasites will be delayed, with consequent worse clinical outcomes and increased risk of death compared to infections with drug-sensitive parasites.

Two studies have recently considered the impacts of ART-R on outcomes after therapy of complicated and severe malaria with intravenous artesunate. In a study in Jinja, Uganda, of 100 children hospitalized with malaria (41% meeting WHO criteria for severe malaria) and treated with intravenous artesunate, ART-R mutations were associated with delayed parasite clearance, but other outcomes did not differ between children infected with PfK13 wild-type or mutant parasites; none of the children died.^11^ However, only 10% of the infections had validated PfK13 ART-R mutations, limiting statistical power. In a more recently published study of 465 children hospitalized for malaria (77% meeting WHO criteria for severe malaria) in northern Uganda and treated with intravenous artesunate (a portion of the SMAART observational study of severe malaria; ISRCTN79071535), prevalence of two validated ART-R mutations, PfK13 C469Y or A675V, was high (combined prevalence of mixed and pure mutant infections 50.3%).^12^ As in the earlier study, children infected with parasites containing ART-R mutations had delayed parasite clearance. However, as in the earlier study infection with ART-R parasites was not associated with worse clinical outcomes. Mortality was actually lower, although not significantly so, in children infected with PfK13 mutant compared to wild-type parasites through day 5 (1.7% vs. 5.5%), day 28 (2.8% vs. 6.6%), or day 180 (3.4% vs. 8.2%). Other clinical outcomes, including length of hospital stay, lactate clearance, transfusion requirement, incidence of subsequent febrile illness or malaria slide positivity by day 28, or incidence of readmission by day 180 did not differ between children infected with PfK13 mutant vs. wild-type parasites. Thus, despite delayed parasite clearance, severe malaria due to ART-R parasites was controlled by artesunate at least as well as severe malaria due to drug-sensitive parasites.

Why weren’t clinical outcomes worse in severe malaria caused by ART-R compared to drug sensitive parasites? One would predict that slower parasite clearance, as seen in ART-R, would have negative clinical consequences, since, without prompt control of infection, severe malaria can progress rapidly to life-threatening complications. However, as malaria parasites and other microbes evolve to circumvent drug pressure, they often undergo alterations in fitness or virulence.^13^ ART-R parasites have decreased uptake of hemoglobin at the young ring stage, limiting activation of artemisinins in the parasite food vacuole.^9^^,^^14^ This alteration leads to delayed clearance of these parasites after treatment with artemisinins, but slowed hemoglobin utilization might also decrease parasite fitness or virulence, allowing those infected with ART-R parasites and treated with artesunate to control the infection at least as well as they do infection with drug sensitive parasites.

The two new studies offer valuable guidance for the management of severe malaria. Previously, anecdotal case reports suggested possible problems with the efficacy of intravenous artesunate in the setting of ART-R.^15^ With uncertainty as to whether ART-R mutations will worsen severe malaria outcomes after treatment with artesunate, some providers are considering a return to quinine to treat severe malaria, and current WHO guidelines state “in areas with established artemisinin resistance, it is recommended that parenteral artesunate and parenteral quinine be given together in full doses”.^16^ However, coadministration of the two drugs will be logistically challenging, and both a clinical trial from Thailand before known emergence of ART-R^17^ and a study in travelers in Italy with imported malaria^18^ showed no benefits and increased adverse events (in some patients necessitating discontinuation of quinine) with combined artesunate + quinine vs. artesunate alone. Results from the two new Ugandan studies suggest that artesunate remains fully efficacious for the treatment of severe malaria in children in areas of Africa with ART-R. Outcomes after treatment with artesunate were as good for PfK13 mutant infections as for wild-type infections, supporting older recommendations to treat severe malaria with intravenous artesunate, followed upon stabilization by a full course of an ACT. However, the drug resistance landscape in Africa is fluid, PfK13 mutations other than C469Y and A675V seen in parts of Africa and elsewhere might be associated with greater loss of artesunate activity, and additional close monitoring of the efficacy of artesunate for the treatment of severe malaria is needed.

## References

[1] Dondorp A Nosten F Stepniewska K Day N White N, 2005. Artesunate versus quinine for treatment of severe falciparum malaria: A randomised trial. Lancet 366: 717–725. 10.1016/S0140-6736(05)67176-0 16125588

[2] Dondorp AM, , 2010. Artesunate versus quinine in the treatment of severe falciparum malaria in African children (AQUAMAT): An open-label, randomised trial. Lancet 376: 1647–1657. 10.1016/S0140-6736(10)61924-1 PMC3033534 21062666

[3] Rosenthal PJ, 2008. Artesunate for the treatment of severe falciparum malaria. N Engl J Med 358: 1829–1836. 10.1056/NEJMct0709050 18434652

[4] Noedl H Se Y Schaecher K Smith BL Socheat D Fukuda MM, 2008. Evidence of artemisinin-resistant malaria in western Cambodia. N Engl J Med 359: 2619–2620. 10.1056/NEJMc0805011 19064625

[5] Dondorp AM, , 2009. Artemisinin resistance in Plasmodium falciparum malaria. N Engl J Med 361: 455–467. 10.1056/NEJMoa0808859 PMC3495232 19641202

[6] Dhorda M Amaratunga C Dondorp AM, 2021. Artemisinin and multidrug-resistant Plasmodium falciparum - A threat for malaria control and elimination. Curr Opin Infect Dis 34: 432–439. 10.1097/QCO.0000000000000766 PMC8452334 34267045

[7] Amaratunga C, , 2016. Dihydroartemisinin-piperaquine resistance in Plasmodium falciparum malaria in Cambodia: A multisite prospective cohort study. Lancet Infect Dis 16: 357–365. 10.1016/S1473-3099(15)00487-9 PMC4792715 26774243

[8] Ariey F, , 2014. A molecular marker of artemisinin-resistant *Plasmodium falciparum* malaria. Nature 505: 50–55. 10.1038/nature12876 PMC5007947 24352242

[9] Rosenthal PJ Asua V Conrad MD, 2024. Emergence, transmission dynamics and mechanisms of artemisinin partial resistance in malaria parasites in Africa. Nat Rev Microbiol 22: 373–384. 10.1038/s41579-024-01008-2 38321292

[10] World Health Organization, 2025. World Malaria Report. Geneva: World Health Organization.

[11] Henrici RC, , 2025. Artemisinin partial resistance in Ugandan children with complicated malaria. JAMA 333: 83–84. 10.1001/jama.2024.22343 PMC11565293 39540797

[12] Maitland K, , 2026. Intravenous Artesunate in Artemisinin-Resistant Severe Malaria in Uganda. N Engl J Med 394: 2380–2382. 10.1056/NEJMc2517274 42234998

[13] Rosenthal PJ, 2013. The interplay between drug resistance and fitness in malaria parasites. Mol Microbiol 89: 1025–1038. 10.1111/mmi.12349 PMC3792794 23899091

[14] Birnbaum J, , 2020. A Kelch13-defined endocytosis pathway mediates artemisinin resistance in malaria parasites. Science 367: 51–59. 10.1126/science.aax4735 31896710

[15] Phyo AP, , 2018. Poor response to artesunate treatment in two patients with severe malaria on the Thai-Myanmar border. Malar J 17: 30. 10.1186/s12936-018-2182-z PMC5769511 29334942

[16] World Health Organization, 2025. WHO Guidelines for Malaria. Geneva: World Health Organization.

[17] Newton PN, , 2001. A comparison of artesunate alone with combined artesunate and quinine in the parenteral treatment of acute falciparum malaria. Trans R Soc Trop Med Hyg 95: 519–523. 10.1016/s0035-9203(01)90025-2 11706665

[18] Botta A, , 2022. Artesunate monotherapy versus artesunate plus quinine combination therapy for treatment of imported severe malaria: A retrospective cohort study. Infection 50: 949–958. 10.1007/s15010-022-01771-5 PMC9338132 35220555

